# The complete chloroplast genome of newly alien medicinal and toxic species, *Zaleya pentandra* (L.) C.Jeffrey (Aizoaceae), in China

**DOI:** 10.1080/23802359.2019.1674709

**Published:** 2019-10-07

**Authors:** Han Xu

**Affiliations:** Institute of Plant Quarantine, Chinese Academy of Inspection and Quarantine, Daxing District, Beijing, China

**Keywords:** Chloroplast genome, *Zaleya pentandra* (L.) C. Jeffrey, Aizoaceae, Caryophyllales, China

## Abstract

The first complete chloroplast genome sequences of new intercepted alien weeds in China, *pentandra* (L.) C. Jeffrey, were reported in this study. The *Z. pentandra* plastome was 153,239 bp long, with the large single copy (LSC) region of 83,528 bp, the small single copy (SSC) region of 18,081 bp, and two inverted repeat (IR) regions of 25,815 bp. The plastome contained 130 genes, including 85 protein-coding, 8 ribosomal RNA, and 37 transfer RNA genes. The overall GC content was 37.0%. Phylogenetic analysis of 32 representative plastomes within the family Aizoaceae suggests that *Zaleya* is more closely related to *Tetragonia* than to *Sesuvium* which is different previous studies. However, due to the limited chloroplast genome accessions in Aizoaceae at present, the phylogenetic relationship between the subfamilies of the family cannot be further discussed.

The genus *Zaleya* Burm. f. consists of about 4 species with accepted names of prostrate annual or perennial herbs (The Plant List 2013), and belongs to family Aizoaceae which consists of about 127 genera and 2500 species (Schweingruber et al. 2011), and is native to tropical Africa, Asia and Australia (eFloras 2008). *Zaleya pentandra* L. is a prostrate perennial herb and the most widely distributed species in the genus with 993 occurrence records (GBIF 2017), and native of Africa and spread to Pakistan and so on (Akbar and Khatoon 2012). In addition, *Z. pentandra* as a medicinal plant can be used for stomach ailment, etc. (Hameed et al. 2011). But it is reported as an invasive weed in Pakistan, and is considered to be a dangerous poison in India (Munawar et al. 2015; Tropical Plants Database 2019). Therefore, with its discovery in trade goods, we should be alert to its harmful risks in China while developing it as a beneficial ingredient of drugs.

Aizoaceae was divided into five subfamilies presented by Bittrich and Hartmann (1988), and Hartmann (1993). *Zaleya pentandra* is subordinate to Sesuvioideae, and closer with *Sesuvium* and *Trianthema* based on three regions (Thiede and Liede-Schumann 2012). At the chloroplast genome level, the taxonomic status of *Zaleya* has not been discussed. Therefore, the chloroplast genome of *Z. pentandra* was analyzed in this paper.

Total DNA (Voucher specimen: 34.747145°N, 119.388760°E, 12183) was isolated using the Plant Genomic DNA Kit (Tiangen Biotech Co., Beijing, China) and sequenced by the Illumina HiSeq2500 platform (Novogene, Beijing, China). A total of 1,354,732 paired-end reads were assembled to *Mesembryanthemum cordifolium* (GenBank accession nr: MK397873) to produce contigs using Geneious assembler (Biomatters, Auckland, New Zealand).

The total plastome length of *Z. pentandra* (MN296416) was 153,239 bp, with a large single copy (LSC; 83,528 bp), small single copy (SSC; 18,081 bp), and two inverted repeats (IRa and IRb; 25,815 bp each). The overall GC content was 37.0% (LSC, 35.0%; SSC, 30.1%; IRs, 42.8%) and the plastome contained 130 genes, including 85 protein-coding, eight rRNA, and 37 tRNA genes. A total of 18 genes were duplicated in the inverted repeat regions including seven tRNA, four rRNA, and seven protein-coding genes.

To confirm the phylogenetic position of *Zaleya*, 32 representative species of Caryophyllales referring to 18 family were aligned using MAFFT v.7.388 (Katoh and Standley 2013) plug-in Geneious Prime v. 2019.1.3 (Biomatters, Auckland, New Zealand) and neighbour-joining (NJ) analysis was conducted with *Berberidopsis corallina* (Berberidopsidales) as an outgroup using Geneious Tree Builder of Geneious Prime v. 2019.1.3 (Biomatters, Auckland, New Zealand) and confidence for nodes determined using bootstrap analysis with 1000 replicates (Figure 1). Unlike previous studies (Thiede and Liede-Schumann 2012), *Zaleya* is more closely related to *Tetragonia* than to *Sesuvium*. However, due to the limited chloroplast genome accessions in Aizoaceae at present, the phylogenetic relationship between the subfamilies of the family cannot be further discussed. In this paper, new contributions of data have been made to the classification study of the Aizoaceae family.

**Figure 1.**
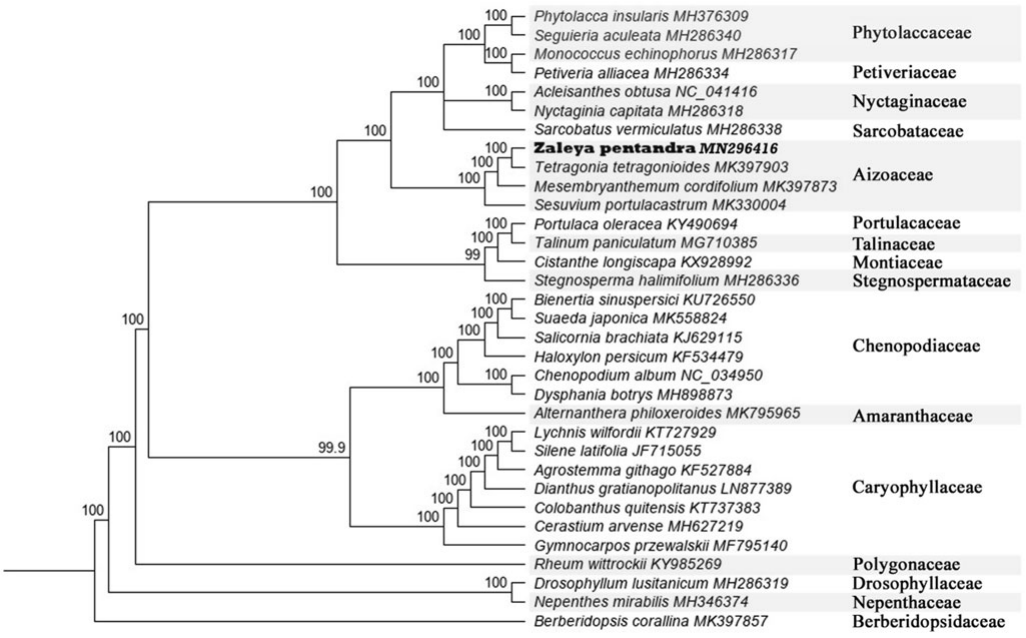
The neighbour-joining (NJ) tree based on the 32 representative chloroplast genomes of order Caryophyllales and one outgroup of Berberidopsidales. The bootstrap value based on 1000 replicates is shown on each node.
